# Adverse effect of inhalational anesthetics on the developing brain

**DOI:** 10.1186/2045-9912-4-2

**Published:** 2014-02-14

**Authors:** Mike Wang, John H Zhang, Richard L Applegate

**Affiliations:** 1 Department of Anesthesiology, Loma Linda University School of Medicine, Loma Linda, CA 92350, USA; 2 Departments of Physiology, Neurosurgery, and Anesthesiology, Loma Linda University School of Medicine, Loma Linda, CA, USA

**Keywords:** Anesthetics, Developing brain, Neurotoxicity

## Abstract

We did a PubMed search and summarized studies on the potential adverse effect of anesthetics especially neurotoxicity in the developing brain, so named anesthesia-induced developmental neurotoxicity. Even though many experimental studies using animal models indicated some adverse effect of anesthetics, more evidence is needed before a recommendation can be made to change the way those anesthetics are used in the pediatric population. Two large clinical trials are underway and may provide insight to the potential human neurotoxic effect of anesthetics.

## Introduction

Each year in the United States, approximately three million infants and children receive anesthesia for surgical procedures, with many more receiving anesthesia for imaging studies or dental procedures [1]. It was previously thought that anesthetic drugs caused short term sedation without any long term sequelae in the developing brain. This notion has now come into question, with numerous animal studies showing that general anesthetics have neurodegenerative effects on the developing brain, causing anesthesia-induced developmental neurotoxicity (AIDN).

In 2000, Ikonomidou et al showed that ethanol, acting by a dual mechanism [blockade of *N*-methyl-D-aspartate (NMDA) receptor and excessive activation of γ-aminobutyric acid (GABA_A_) receptors], triggers diffuse neuroapoptosis in the developing rat brain, with the peak of toxicity coinciding with the period of synaptogenesis [2]. These results raised questions of whether general anesthesia can also cause neuroapoptosis, since like ethanol, most anesthetic medications work as NMDA receptor antagonists or GABA_A_ receptor agonists.

Subsequently in 2003, Jevtovic-Todorovic et al demonstrated that a one time exposure to a common anesthetic cocktail of isoflurane, midazolam, and nitrous oxide, also caused neuroapoptosis in the developing rat brain during the period of synaptogenesis [3]. This one time anesthetic exposure was also found to cause long term neurocognitive dysfunction that continued from adolescence into adulthood. These results in an animal model again raised questions of whether a routine anesthetic exposure could cause AIDN in neonates, infants, or even very young children.

These findings caused much debate within the pediatric anesthesia community at the time they were published. Many counter points were raised that highlighted the differences between anesthetic exposure in rats and humans [4]. It was argued that there is less control over environmental conditions when anesthesia is performed on rats, with hypoxemia, hypercarbia, and hypoglycemia being potential confounding variables in lab experimentation. When subsequent publications in animal models showed evidence of AIDN despite controlling for physiological variables [5,6], the focus of research shifted to determine what ramifications these results might have on the clinical practice of pediatric anesthesia. In March of 2007 the US Food and Drug Administration held an advisory meeting to review the data on AIDN and decide whether a change in pediatric anesthesia practice was warranted [7].

Though the advisory meeting did not recommend making any formal changes to current pediatric anesthesia practices, it did urge the anesthesia community to conduct studies to determine if AIDN can occur in children. There are currently two large prospective multi-center trials being conducted. The Pediatric Anesthesia NeuroDevelopment Assessment (PANDA Study) [8] is looking at children exposed to a single general anesthetic before age three compared to a control sibling who has not had anesthesia. The second trial is the GAS study (NCT00756600), comparing spinal or general anesthesia for inguinal hernia repair in newborns. Due to the nature of these trials, it will still be a number of years before any meaningful data on neurodevelopmental outcomes will be published. Thus it is prudent to continue conducting and reviewing new developments in animal research on AIDN.

## Methods

A PubMed search was done in April 2013 with search terms in Table 1. Intravenous anesthetic agents were included as search terms to obtain a broader range of articles, even though they are not covered in this review. In addition to this database search, reference lists of relevant articles were reviewed for additional publications of interest.

**Table 1:** Search terms used in review article database search

**Database**	**Search terms**
PubMed	Brain (newborn or infant or child or neonate or neonatal or animals, newborn) and (neurodegeneration or apoptosis or toxicity or neurocognitive impairment or developmental impairment or developmental disabilities, or learning disorders) and (isoflurane or desflurane or sevoflurane or propofol or etomidate or ketamine or lorazepam or diazepam or midazolam or pentobarbital or phenobarbital or anesthesia, IV or anesthesia, inhalation or anesthesia).
	In PubMed, terms are searched as subject headings and keywords simultaneously.
	Articles were limited to those printed or translated into English.

## Review

The PubMed search resulted in 347 articles, of which 84 articles were identified as pertaining to the topic. Out of this group of articles, a subset of 44 articles was identified as being relevant to inhalational anesthetics and their effects on the developing animal brain.

### Inhalational anesthetics

In the pediatric population, inhalational anesthetics are by far the most common drugs used for the induction and maintenance of general anesthesia. Among the articles reviewed, inhalational anesthetics have been shown to be neurotoxic in the developing brain of all animal models tested to date, which include rats, mice, guinea pigs, piglets, and rhesus monkeys [3,6,9-13]. Mechanistically speaking, inhalational anesthetics cause general anesthesia predominately as GABA_A_ agonists [14] and NMDA receptor antagonists [15], though there are varying degrees of affinity for these receptors among differing anesthetic medications. These differences might give insight into why certain inhalational anesthetics cause more neurodegeneration than others.

### Isoflurane

Since 2003, isoflurane has been the most extensively studied inhalational anesthetic. In an attempt to create clinically relevant animal models, a number of studies have looked into the minimum alveolar concentration (MAC) of isoflurane in newborn rodents. It has been established that the MAC of 2.5 month old rats does not change depending on the length of anesthesia if physiologic parameters are kept constant [16]. More recently it was shown that the MAC of isoflurane in nine day old rats (P9) is 2.34% [17], which was the basis for isoflurane concentrations used in studies of developmental neurotoxicity. The notion of a static MAC value has now come into question for neonatal rodents.

Stratmann et al in a study on P7 rats found that MAC requirements decreased from 1 h to 4 h into an anesthetic [18]. With direct sampling of brain partial pressures of isoflurane, this decrease in MAC was found to come about in P7 rats even after full equilibration with inspiratory gas concentration, suggesting a pharmacodynamic process that occurs in P7 rats but not P60 rats. In P7 rats median MAC was 2.75% at 1 h and 1.3% at 4 h of isoflurane anesthesia, while in P60 rats median MAC was 1.65% at 1 h and 1.5% at 4 h. Similar decreases in MAC with increased duration of anesthesia have also been documented in neonatal mice, using isoflurane, sevoflurane or desflurane as a sole anesthetic agent [19]. More research is needed to see if this phenomenon of decreasing MAC requirements in neonatal animal models has any bearing on clinical practice.

In light of this new finding, studies on AIDN in animal models can be reexamined in regards to dosages chosen. In P7 rats, a combination of 0.75% isoflurane with 9 mg/kg midazolam and 75% nitrous oxide for 6 h caused widespread AIDN followed by learning impairment at P32 that lasted until adulthood at P131 [3]. However, this anesthetic exposure did not have any effects on overall growth, sensory motor ability, spontaneous locomotion, or attention. Using a MAC of 2.21 atm for nitrous oxide in Sprague-Dawley rats [20], the combined MAC of the anesthetic gases at 1 h exposure totaled 0.61 MAC, and at 4 h exposure totaled 0.92 MAC. These MAC levels are similar to those used in everyday clinical practice.

The same combination of anesthetic agents exposed to rats aged P1 to P14 for 6 h, again showed evidence of AIDN in all age groups tested, with the height of toxicity at age P7, coinciding with the peak of synaptogenesis [6,21]. Similar studies in guinea pigs [10], piglets [12], and rhesus monkeys [11] all showed age dependent vulnerability to AIDN, demonstrating that AIDN exists in animals with longer periods of synaptogensis like humans. Rodent in vitro studies have also shown comparable results [5,22]. A similar time period of peak synaptogenesis in humans is thought to range from the third trimester of gestation to several years after birth [21]. It remains to be seen whether AIDN occurs in humans, and if it does, the exact age of maximal vulnerability to anesthetic agents.

Other animal studies that have shown declines in neurocognitive function after a single isoflurane exposure include an experiment on P7 rats exposed to 0.75% isoflurane and 70% nitrous oxide for 6 h [23]. These rats showed long term memory impairment at P47 when assessed with trace fear conditioning. In the same study, pretreatment with 70% xenon for 2 h prior to isoflurane and nitrous oxide exposure was able to attenuate AIDN. Xenon pretreatment rats did not differ compared to control rats when long-term memory was assessed at P47. In a follow up study, the same anesthetic regimen was exposed to P7 rats, causing AIDN and long-term memory impairment at P47 [24]. A single 0.3 cm surgical incision made to the left hind paw at the start of the anesthetic exposure increased neuroapoptosis rates by approximately 60% compared to anesthesia alone, and statistically increased the degree of long term memory dysfunction when compared to anesthesia alone at P47. However, although P7 mice exposed to isoflurane had apoptotic cell death early after exposure, no differences in adult cell density, learning or activity was found in isoflurane-exposed compared to controls [25].

Of interest is an article published April 2013, in which isoflurane was shown to be neuroprotective against AIDN. In this study, hippocampal slice cultures of P7 rats were exposed to 1 or 2 MAC of xenon, isoflurane, or sevoflurane [26]. All three anesthetics caused similar levels of AIDN in this in vitro study. Preconditioning with 1.4% isoflurane (0.75% MAC) for 2 h attenuated neuroapoptosis to control levels. More research is needed to see if this neuroprotective effect of isoflurane can be shown to reverse AIDN induced neurocognitive disfunction in in vivo animal models.

### Sevoflurane

Sevoflurane is the most commonly used inhalational anesthetic in the USA for pediatric surgical cases. Due to its minimal airway reactivity and low blood/gas partition coefficient, sevoflurane has quickly become the inhalational induction agent of choice in operating rooms nationwide. Despite being so commonplace in pediatric anesthesia practice, the number of sevoflurane specific AIDN studies is small in comparison to isoflurane. The studies that have been published on sevoflurane and AIDN suggest a similar neurotoxic effect when compared to isoflurane administration in animal models.

In 2008 Zhang et al published the data on sevoflurane and AIDN. The study consisted of P7 mice exposed to 2 h of 1.7% sevoflurane anesthesia, resulting in significant AIDN in the sevoflurane group as compared to control using activated capase-3 analysis [27]. Recently published MAC data of 3.8% at 1 h and 3.3% at 3 h of sevoflurane exposure [18], suggests that the concentration of 1.7% sevoflurane correlates to 0.45-0.52 MAC, which is a subclinical dose. A subsequent study exposed P6 mice to 3% sevoflurane for 6 h, showing wide spread AIDN, long term memory deficit from 8 weeks to 14-17 weeks of age as assessed with contextual/cued fear testing, and abnormal social interaction at age of 18 weeks of age, showing that sevoflurane like isoflurane causes long lasting neurocognitive dysfunction after a one time exposure in an animal model [28]. In contrast, in a study that compared isoflurane to sevoflurane anesthesia exposure in P7 rats, although markers of apoptosis were greater after isoflurane, neither agent was associated with impaired learning or memory when tested 31 to 40 days after anesthesia exposure [29].

Various case reports [30] as well as several studies [31,32] have demonstrated epileptiform electroencephalogram and seizure activity during induction with sevoflurane in humans, while other studies have not reported these results [33,34]. In 2010 Edwards et al reported that 40% of rats aged P4 to P8 developed distinct episodes of epileptic seizures during maintenance with 2.1% sevoflurane [35]. These seizure-like episodes were not found in P10 to P17 rats during maintenance of anesthesia. Emergence after 3 h of sevoflurane anesthesia caused some tonic/clonic seizures in P10 to P17 rats, but not P4 to P8 rats. Bumetanide pretreatment significantly decreased the seizure activity in P4 to P8 rats during maintenance anesthesia, but not P10 to P17 rats during emergence. The same study also showed that exposure of P4 rats to 2.1% sevoflurane for 6 h caused significant AIDN. This effect was attenuated and reduced to control levels by pretreatment with bumetanide 15 minutes prior to sevoflurane exposure.

Two novel approaches to neuroprotection against AIDN caused by sevoflurane were recently published. The first study exposed P6 mice to 3% sevoflurane for 6 h with or without 1.3% hydrogen as part of the carrier gas [36]. The concentrations of hydrogen gas used in this study were low enough to avoid explosion [37,38]. The mice exposed to sevoflurane alone showed significant increases in neuroapoptosis. Neurocognitive testing showed no different in general behavior or short term memory at 12 weeks of age, but did demonstrate a deficit in long term memory at 13 weeks of age. Coadministration of hydrogen gas during the sevoflurane anesthetic significantly reduced the extent of neuroapoptosis, and suppressed the impairment in long-term memory seen in mice exposed to sevoflurane alone. Free oxygen radical scavenging, one proposed effect of hydrogen gas administration, prevented cognitive decline in P7 rats exposed to general anesthesia [39]. The second study tested the effect of environmental enrichment on AIDN. Environmental enrichment has been shown to increase learning and memory after traumatic brain injury [40-42]. Pregnant mice at gestational day 14 (G14) were exposed to 2.5% sevoflurane for 2 h, which in turn caused significant increases in neuroapoptosis in fetal mice [43]. Offspring mice were delivered at G21 and exposed to either standard environment or environmental enrichment. Mice in the standard enrichment group were shown to have impaired learning and memory at age P31 to P37 as assessed by the Morris water maze. Mice in the environmental enrichment group did not have impairment in learning or memory as compared to the control group, demonstrating that environmental enrichment is able to mitigate neurocognitive dysfunction caused by AIDN in an animal model.

### Desflurane

Being one of the newest inhalational anesthetics used in clinical practice, it’s not suprising that desflurane has the least amount of published data in regards to AIDN. In 2011 two animal studies were published on comparative neurotoxicity of desflurane, sevoflurane, and isoflurane, bringing into question whether inhalational anesthetics cause similar levels of neurodegeneration at equivalent MAC values.

The first comparison study exposed P7-8 mice to 6 h of 7.4% desflurane, 2.9% sevoflurane, or 1.5% isoflurane, which resulted in similar levels of AIDN as assessed immunohistochemically and by colorimetric caspase 3 assay [44]. A second study published later in the year had a very similar study design, where P6 mice were exposed to 6 h of 8% desflurane, 3% sevoflurane, or 2% isoflurane, showing that 8% desflurane caused more neurodegeneration than 2% isoflurane, which caused more toxicity than 2% sevoflurane [18]. In the same study, behavioral testing showed that mice in the desflurane group had impaired working memory at week 6 of age as assessed by Y-maze, and impaired long term memory at week 7 of age as assessed by fear conditioning. The mice in the sevoflurane and isoflurane groups did not show impaired working memory, but did have impaired long term memory, which would further support the notion of desflurane being more neurotoxic than sevoflurane or isoflurane at equivalent MAC doses.

More studies need to be conducted to better determine if inhalational anesthetics have different neurotoxic profiles at equivalent MAC doses in animals, as current studies have conflicting results.

### Nitrous oxide

Interestingly, nitrous oxide is the only inhalational anesthetic that has not caused AIDN in animal models when used as a sole anesthetic [45]. When P7 Sprague-Dawley rats were exposed to 50%, 100%, or 150% (in a hyperbaric chamber) nitrous oxide for 6 h, no significant increases in neuroapoptosis were noted [3,6]. However, 75% nitrous oxide added to 0.75% isoflurane worsened AIDN compared to isoflurane alone, suggesting that nitrous oxide does have an additive toxicity effect when combined with other anesthetic agents. Unlike xenon, pretreatment with nitrous oxide before an anesthetic exposure does not attenuate levels of neurodegeneration [22].

### Xenon

Xenon is an interesting anesthetic gas in regards to animal studies and AIDN. The earliest animal study on xenon and AIDN showed that P7 Sprague-Dawley rats exposed to 75% xenon for 6 h did not have any significant increase in neurodegeneration, and that xenon when added to 0.75% isoflurane for 6 h attenuated AIDN in a dose dependent manner [46]. A repeat study, this time in P7 mice, again showed that xenon is able to decrease levels of AIDN when added to 0.75% isoflurane for 4 h [9]. However in the same study, 70% xenon for 4 h as a sole anesthetic caused significant increases in neurodegeneration, which raised the question of whether xenon was as benign as suggested by the prior study in P7 rats.

A study on hippocampal slice cultures from P7 rats concluded that xenon increased neuroapoptosis in a similar fashion to sevoflurane and isoflurane at equipotent concentrations [24]. Xenon at 0.75 MAC (60% at 1.2 atm) for 6 h did not show any significant increase in AIDN. However, higher doses of xenon at 1 and 2 MAC (60% at 2.67 or 3.67 atm) for 6 h did show significant increases in neurodegeneration. Interestingly, pretreatment with 1.4% isoflurane (0.75 MAC) for 2 h, followed by a 6 h exposure to 1 MAC of either xenon, isoflurane, or sevoflurane 26 h after pretreatment, was associated with attenuation of AIDN compared to groups without pretreatment. In vivo studies are needed to confirm whether this pretreatment strategy is effective in live animals.

## Discussion

In 2003 the results on AIDN published by Jevtovic-Todorovic et al [3] created much debate within the pediatric anesthesia community. After a decade of subsequent research, it is evident that in multiple differing mammalian species, exposure to anesthetic medications during a period of brain vulnerability (peak synaptogenesis) is associated with significant neurodegeneration and long term neurocognitive dysfunction that lasts into adulthood [3,6,9-13]. Of particular interest is the recent study comparing desflurane, sevoflurane, and isoflurane at equipotent MAC levels [18]. Further investigation into the specific mechanistic differences between these inhalational anesthetics may shed more light on AIDN and how it impairs working and long term memory in mice.

Out of all inhalational anesthetics reviewed, nitrous oxide was the only agent that did not cause AIDN when used as a sole anesthetic agent [3,6]. This key difference may be due to the fact that nitrous oxide exerts its primary effect through NMDA receptor antagonism, instead of GABA_A_ receptor activation. In a similar fashion xenon works predominantly through NMDA receptor antagonism, but has been shown to cause significant increase in neurodegeneration when used as a sole anesthetic [9,24]. Cross comparison study of these two anesthetic agents may further our understanding of how anesthetic agents cause differing levels of AIDN even when they exert their function through the same primary receptor. More research needs to be conducted to determine the exact ramifications these findings should have on the clinical practice of pediatric anesthesiology.

To date there have only been a few observational studies done in humans, which have had mixed findings. A group of studies from the Mayo Clinic have shown that multiple anesthetic exposures in infants and children, but not a single exposure, increases the risk of learning disabilities as well as later development of attention-deficit/hyperactivity disorder [47-49]. Similar studies have demonstrated that a single exposure to general anesthesia causes increased risk of developmental disorders and deficits in language/abstract reasoning in children less than 3 years of age [50-52]. Other studies do not find any association between exposure of children to general anesthesia and the development of abnormal behavior or poor academic performance later on in life [53-55]. In addition, two studies on neonates report that exposure to prolonged sedation is not associated with increased risk of abnormal neurodevelopment [56,57].

The aforementioned human observational studies all used databases that were originally collected for purposes other than research on AIDN. Observational studies have significant limitations due to confounding variables, which make interpretation of these studies extremely complicated. The lack of prospective trials specifically focused on AIDN and the developing human brain makes it difficult to recommend if any changes should be made to the way anesthesia is currently practiced in infants and children.

## Conclusion

At this time the anesthesia community is waiting for guidance from prospective trials such as the PANDA and GAS studies to show whether present clinical practices need to be altered. This is particularly critical, as many pediatric patients have undergone surgery and anesthesia with no apparent harm. Further it may be difficult to distinguish the effects of anesthesia on the developing brain from surgery-related effects that may follow the condition that mandated surgery or postoperative events such as inflammation, pain and any necessary drug treatments. Well-designed prospective studies investigating AIDN in humans may take years to reach conclusions, thus it is prudent to continue research in animal models, particularly focusing on determining the specific mechanistic pathways that cause AIDN. Studies that identifiy mechanistic pathways resulting in AIDN may then provide insight into novel treatment modalities that can hinder or reverse causes of neurodegeneration.

## Abbreviations

AIDN: Anesthesia-induced developmental neurotoxicity; NMDA: *N*-methyl-D-aspartate; GABAA: γ-aminobutyric acid receptor type A; MAC: Minimum alveolar concentration.

## Competing interests

The authors declare they have no competing interests.

## Authors’ contributions

MW designed the review, carried out the literature search, analyzed the included literature and drafted the manuscript. JZ helped in the design of the review, and critically edited the manuscript. RA analyzed the included literature and critically edited the manuscript. All authors read and approved the final manuscript.
